# Author Correction: Reconstruction of human dispersal during Aurignacian on pan-European scale

**DOI:** 10.1038/s41467-025-61311-1

**Published:** 2025-06-26

**Authors:** Yaping Shao, Christian Wegener, Konstantin Klein, Isabell Schmidt, Gerd-Christian Weniger

**Affiliations:** 1 Institute for Geophysics and Meteorology, University of Cologne, Cologne, Germany; 2 Institute of Prehistory, University of Cologne, Cologne, Germany

**Keywords:** Palaeoclimate, Archaeology, Ecological modelling, Population dynamics

Correction to: *Nature Communications* 10.1038/s41467-024-51349-y, published online 28 August 2024

In the version of the article initially published, the “Essen-Fischlaken” site was incorrectly included in the Results and discussion section, Fig. 2 and Table 1. This site has now been removed from the article along with original reference 72. These corrections have been made to the HTML and PDF versions of the article.

The authors would like to thank Michael Baales, Tilman Böckenförde, Gianpiero Di Maida, Dirk Leder and Thomas Terberger for bringing this error to their attention.

