# Supplementary material for: Reconstruction of human dispersal during Aurignacian on pan-European scale
Source: Nat Commun. 2024 Aug 28;15:7406. doi: 10.1038/s41467-024-51349-y (PMC11358479; doi:10.1038/s41467-024-51349-y)
Supplement: Supplementary file 1 — Supplementary Information [file 41467_2024_51349_MOESM1_ESM.pdf]

Supplementary to "Reconstruction of Human Dispersal during Aurignacian on Pan-European Scale"

Yaping Shao[1], Christian Wegener[1], Konstantin Klein[1], Isabell Schmidt[2] and Gerd-Christian Weniger[2]

[1]Institute for Geophysics and Meteorology, University of Cologne, Albertus-Magnus-Platz 1, Cologne, 50923, Germany.
[2]Institute of Prehistory, University of Cologne, Albertus-Magnus-Platz 1, Cologne, 50923, Germany.

Contributing authors: yshao@uni-koeln.de;

1 Supplementary Figures

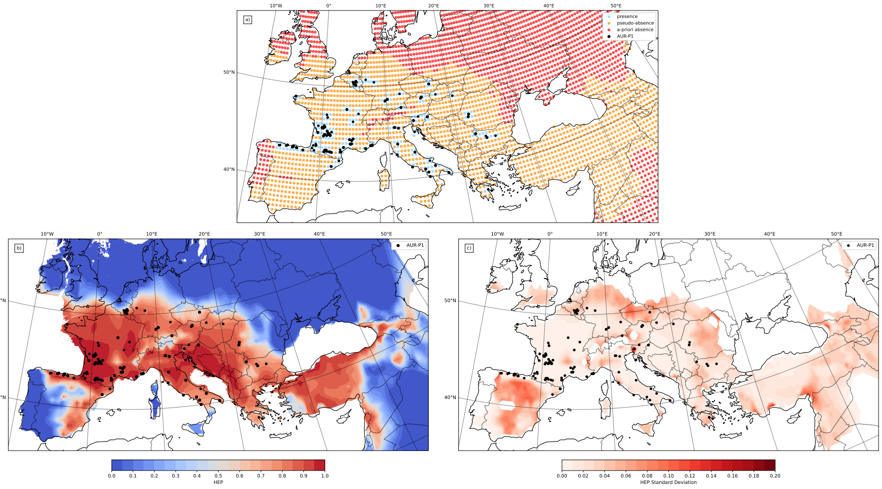

Supplementary Fig. 1 Steps of HEP creation. a): Illustration of spatial blocking of the archaeological site data for training of the HEP model. Shown are a-priori absence cells (red), pseudo-absence cells (orange), presence cells (cyan) and the locations of the AUR-P1 archaeological sites (full black dots); b) Model-simulated HEP for interstadial period of AUR-P1; and c) Standard deviation of the model-simulated HEP, estimated from the 1000 model-training runs.

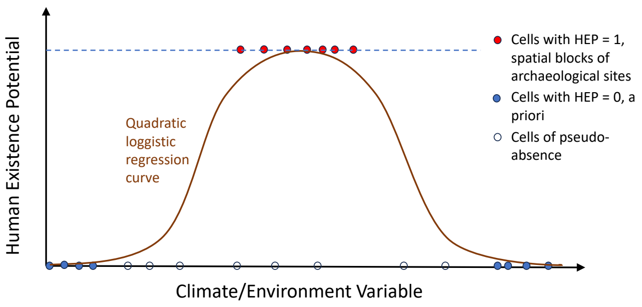

Supplementary Fig. 2 Illustration of quadratic logistic regression. The HEP values for pseudo-absence cells are estimated using the logistic-regression model.

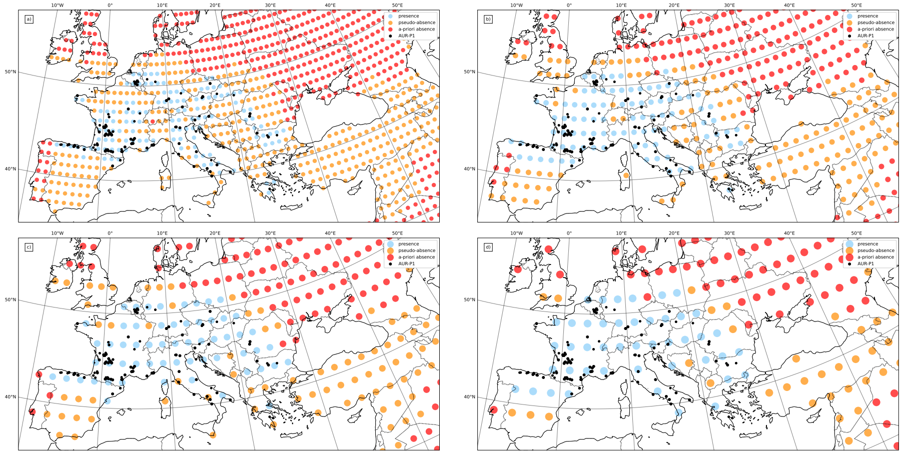

Supplementary Fig. 3 Investigation of impact of spatial blocking on HEP estimates. a) Distribution of presence, a-priori-absence and pseudo-absence cells for spatial-block size of 1.0° for AUR-P1 interstadial case; b) As a), but for spatial-block size of 1.5°, c) As a), but for spatial-block size of 2.0°; and d) As a), but for spatial-block size of 2.5°.

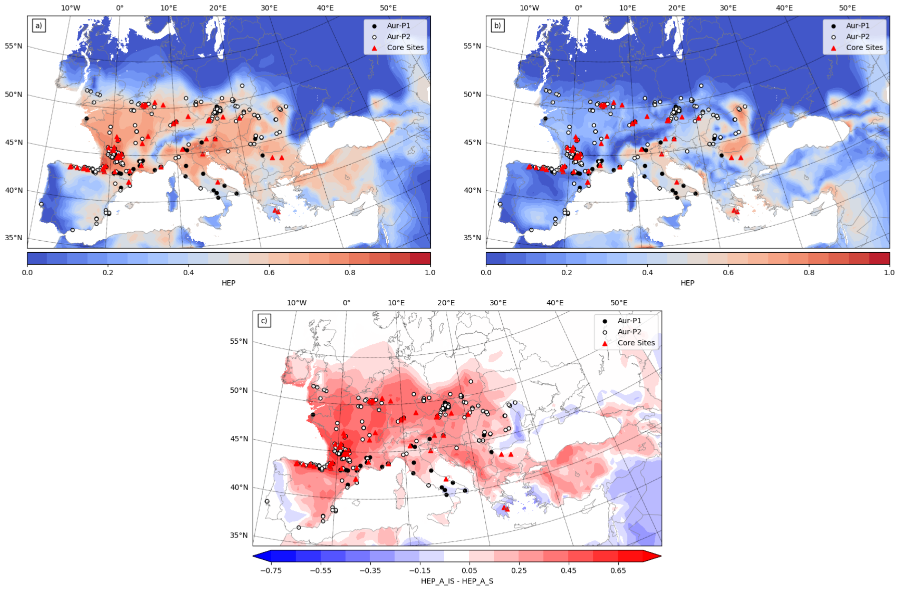

Supplementary Fig. 4 Accessible HEPs from different climate conditions. a) Example of accessible human existence potential Φ_{Ac} for proto-typical interstadial times during the AUR. b) as a), but for stadial times; c) HEP difference between interstadial and stadial (interstadial minus stadial). Redrawn from Shao et al[1].

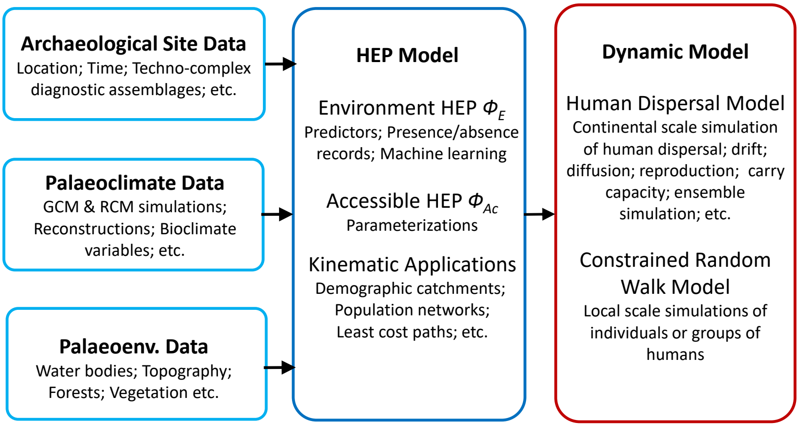

Supplementary Fig. 5 A schematic summary of the OWM framework.

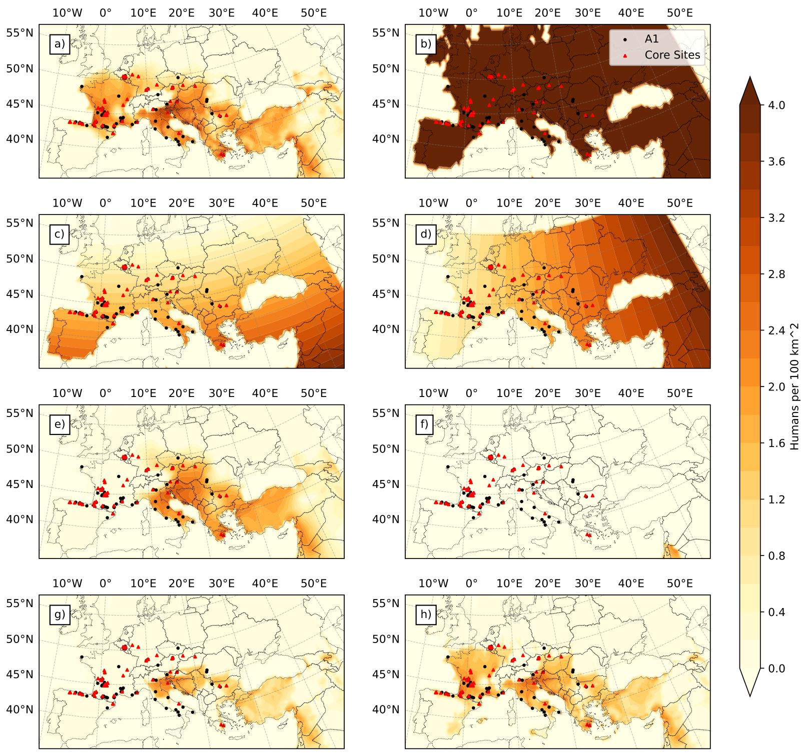

Supplementary Fig. 6 Simulated population density for 41 ka of the experiments listed in Supplementary Table 2. a) full model results as in Fig. 1c; b) as a), but HEP is assumed to be one and uniform on land of the entire domain; c) as b), but HEP is assumed to have a uniform gradient pointing from north to south; d) as c), but HEP is assumed to have an uniform gradient from west to east; e) as a), but the drift term is set to zero; f) as a), but the diffusion term is set to zero; g) as a), but the reproduction term is set to zero and the initial population size is 10 times larger; and h) as a), but with reduced birth rate. The results shown in b) - h) correspond to the numerical experiments BS1 - BS7 listed in Supplementary Tab. 2, respectively.

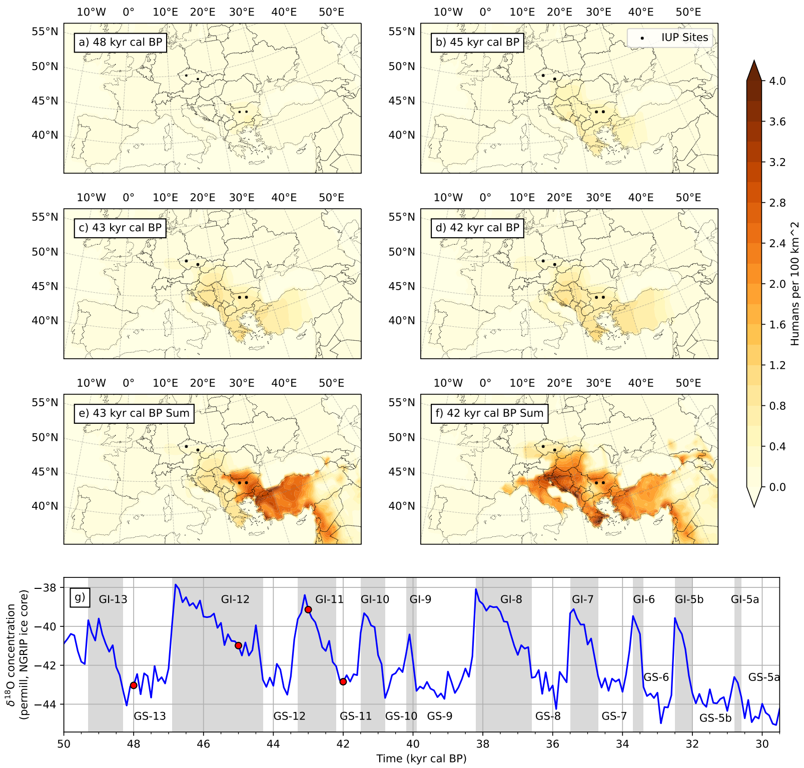

Supplementary Fig. 7 IUP Simulations (BS8). Shown in a) - d) are the simulated IUP population densities for 48, 45, 43 and 42 ka, respectively, with initial population densities specified at 50 ka at four IUP sites marked by the black dots. Shown in e) and f) are the IUP and AUR combined population density for 43 and 42 ka, respectively. The δ^{18}O time series is shown in g). The times, for which population density is shown, are marked with red dots. Shaded Areas correspond to interstadial (GI) and non-shaded to stadial (GS) times.

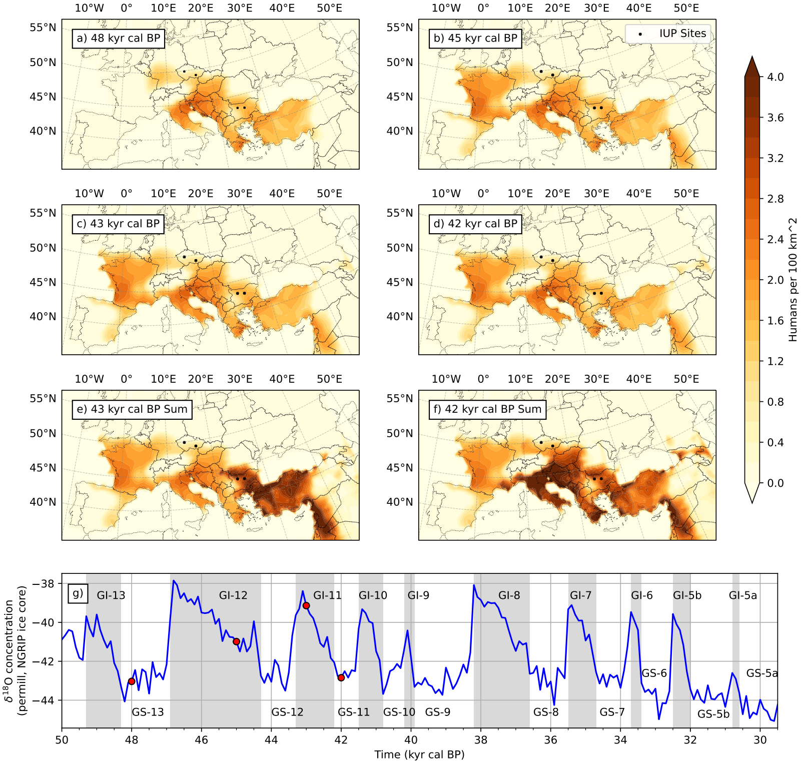

Supplementary Fig. 8 IUP Simulations (BS9). As Supplementary Fig. 7, but with all parameters as for AUR-P1. Shown in a) - d) are the simulated IUP population densities for 48, 45, 43 and 42 ka, respectively. Shown in e) and f) are the IUP and AUR combined population density for 43 and 42 ka, respectively. The δ^{18}O time series is shown in g). The times, for which population density is shown, are marked with red dots. Shaded Areas correspond to interstadial (GI) and non-shaded to stadial (GS) times.

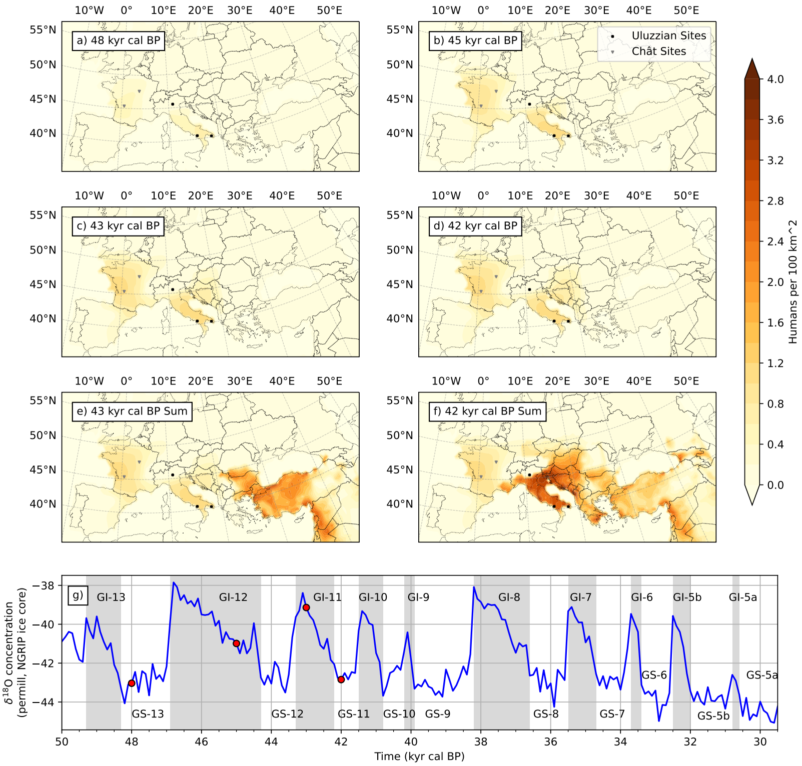

Supplementary Fig. 9 IUP Simulations (BS10). As Supplementary Fig. 7, but with initial population densities specified at 50 ka at three Uluzian sites in Italy (Fumane, Grotta di; Cavallo, Grotta del; and Castelcivita) and two Châtelperronian sites in France (Grotte du Renne; Trou de la Chèvre), as listed in Supplementary Tab. 3. Shown in a) - d) are the simulated IUP population densities for 48, 45, 43 and 42 ka, respectively. Shown in e) and f) are the IUP and AUR combined population density for 43 and 42 ka, respectively. The δ^{18}O time series is shown in g). The times, for which population density is shown, are marked with red dots. Shaded Areas correspond to interstadial (GI) and non-shaded to stadial (GS) times.

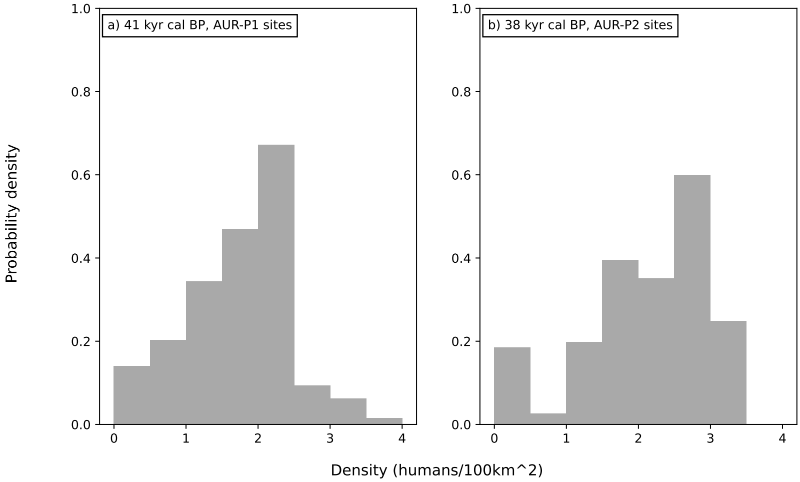

Supplementary Fig. 10 Population density comparison. a) Probability density function of the simulated population density at AUR-P1 sites for 41 ka, and b) as a), but at AUR-P2 sites for 38 ka.

2 Supplementary Tables

Supplementary Table 1 Spatial correlation simulation overview. Brier Skill Score (BSS) and Area under a Receiver (AUC) Operating Characteristics Curve (Area under Curve) for the HEP predictions using the predictors estimated for the AUR interstadial climate. All values are averaged over the 1000 training runs, each using 80% of randomly selected presence/absence records of the AUR-P1 for training and the remaining 20% for validation. For training and validating the HEP model, several spatial-block sizes have been tested, as listed in the Column 2 of the table. For the spatial-block sizes, the numbers of the presence, a-priori-absence and pseudo-absence cells differ, as listed in column 3, 4 and 5. The numbers of presence, a-priori-absence and pseudo-absence cells are denoted with N_p, N_{apa} and N_{pa}, respectively.

Run	Block Size	N_p	N_{apa}	N_{pa}	AUC	Brier-Score
Base	0.5°	173	2445	623	0.920	0.612
/2	1.0°	115	587	135	0.911	0.684
/3	1.5°	96	245	50	0.922	0.671
/4	2.0°	64	141	25	0.892	0.575
/5	2.5°	54	88	11	0.923	0.676

Supplementary Table 2 List of experiments with simple model configurations.

Exp.	Modell	Initial Field	HEP
BS1	Full	Lavent, 45 ka	Uniform HEP = 1
BS2	Full	As above	Uniform N-S gradient; HEP = 1 at 26°N and 0 at 60°N
BS3	Full	As above	Uniform E-W gradient; HEP = 1 at 49°E and 0 at 15°W
BS4	No drift	As above	HEP model
BS5	No diffusion	As above	HEP model
BS6	No reproduction	As above; initial population 10 times larger	HEP model
BS7	Full; reduced birth rate	Random	HEP model
BS8	Full; see text for details	4 IUP sites, 50 ka	HEP model AUR-P1
BS9	Full; see text for details	4 IUP sites, 50 ka	HEP model AUR-P1
BS10	Full; see text for details	3 Uluzian and 2 Chât sites, 50 ka	HEP model AUR-P1

Supplementary Table 3 List of sites considered in the IUP experiments.

Culture	Site	Long.	Lat.	Country
IUP	Bacho Kiro	42.95	25.43	Bulgaria
IUP	Temnata Dupka	43.17	24.06	Bulgaria
IUP	Bohunice	49.17	16.58	Czechia
IUP (?)	Zlatý kůň	49.92	14.07	Czechia
Uluzzian	Fumane, Grotta di	45.55	10.88	Italy
Uluzzian	Cavallo, Grotta del	40.16	17.96	Italy
Uluzzian	Castelcivita	40.49	15.23	Italy
Châtelperronian	Grotte du Renne	47.60	3.74	France
Châtelperronian	Trou de la Chèvre	45.32	0.59	France

3 Supplementary Discussion

3.1 Model Uncertainty and Ensemble Simulation

3.1.1 Idealized Experiments

The OWM is rather complex, consisting of numerical modules for human dispersal and population growth, various archaeological, paleoclimate and palaeoenvironment datasets as input as well as model initialization. To better understand the model results and uncertainties, the OWM performance is first examined using simple configurations. A large number of the numerical experiments have been carried out as reported by Wegener[2], and in Supplementary Fig. 6, the results from a subset of the experiments (listed in Supplementary Table 2) are presented.

Plotted in Supplementary Fig. 6b - h are the simulated population density for 41 ka from the numerical experiments BS1 to BS7. As reference, the results from the full-model run are shown in Supplementary Fig. 6a (identical to Fig. 1c). Supplementary Fig. 6b, c and d show the results generated using simple and stationary HEP patterns. Supplementary Fig. 6b shows the situation of a uniform HEP (= 1) over the entire simulation domain, while Supplementary Fig. 6c and d the situations of a north-south HEP gradient (HEP = 1 at 26°N and 0 at 60°N) and an east-west HEP gradient (HEP = 1 at 49°E and 0 at 15°W). These experiments reveal that, in case of stationary HEP, the population density will reach (in less than one thousand years) a steady state which fulfils the human existence potential, as expected.

Supplementary Fig. 6e shows the results of the numerical experiment BS4, in which the drift term in Eq. (4) is omitted and human dispersal is modelled as a diffusion/reaction process, as in earlier studies[3]. A comparison with Supplementary Fig. 6a reveals that diffusion/reaction is indeed an important process responsible for human dispersal, but the dispersal speed is much lower than expected and the population pattern shows much less structures. In contrast, Supplementary Fig. 6f shows the results of a simulation with no diffusion (BS5). In this case, no human expansion is possible, as drift is always pointed to the centre of high available HEP and regions with no humans have zero

available HEP. Supplementary Fig. 6g shows the result of a simulation with no birth and death (BS6), limiting the total population to a constant (which is initially set to 28000, 10 times that for the full-model run). A comparison with Supplementary Fig. 6a shows that population growth is a major driver for human dispersal. Supplementary Fig. 6h shows the results of a simulation with a low initial population density (between 0 and 0.01 P km^{-2} distributed randomly in the domain) and a low birth rate of 0.1% (BS7). The population pattern seen in Supplementary Fig. 6h is qualitatively similar to that seen in Supplementary Fig. 6a. This shows that the initial population has little impact of the population pattern several thousand years later.

3.1.2 IUP Experiment

As pointed out in Section 1 Introduction and shown by the model results for the Aurignacian, human dispersal is a convoluted process of advance, retreat, abandonment, and resettlement on different scales. In Europe, before the "sudden flowing"[4] of the Aurignacian in the Upper Paleolithic, AMHs of the IUP technologies already existed. Limited skeletal evidence dated to the IUP and AUR-P1 time frame suggests that these earlier AMHs had recent NEA ancestry. It cannot be ruled out that the demographic assimilation of the NEA populations and their replacement were taking place during this period[5]. It is thus appropriate to ask whether the NEA and IUP humans impacted on the dispersal of humans of the Aurignacian in Europe. While the prevailing hypothesis for the Aurignacian emergence in Europe has been a dispersal started somewhere in Turkey[4] and a colonization of the European continent with little resistance, it is conceivable that the Aurignacian settlement in Europe took place through the adoption and expansion of the existing IUP exchange networks.

The possible NEA and AMH interactions at the time of Aurignacian arrival in Europe are very much tangled and hugely uncertain. Bergstrom et al.[6] reported that NEAs contributed about 2% to the genetic signals of the modern humans, suggesting that the interactions between NEAs and AMHs did take place, but the scale of the interactions was limited. Also, the nature, timing and intensity of the interactive processes might have been regionally different. At the time as AMHs of the Aurignacian appeared in Europe, the NEA population was already under decline, if not already extincted. Even for Iberia, which has long been considered to be the last refuge of the NEAs, the timing of NEA extinction is highly debatable. The NEA fossils from El Sidron are dated directly to 48.4 pm 3.2 ka cal BP[7]. Recent analysis suggests that out of the nearly 100 MP sites on the peninsula from the Marine Isotope Stage 3, only three might belong to the period of AUR-P1. Most of the sites dated to the late MP in Iberia suggests an earlier abandonment by the NEAs, probably before 45 ka. This indicates a decline of the NEA population already in Heinrich Event 5 and the eventual disappearance at the latest before Heinrich Event 4. The analysis of Klein et al.[8] concluded that a significant population overlap between NEAs and AMHs of AUR in Iberia is unlikely. Based on these

considerations, it is plausible to assume that the impact of the NEAs on the Aurignacian dispersal is small.
The IUP population size in Europe at the time when humans of the AUR appeared is not well known. The climate from about 47 to 43.5 ka was cooling, reaching the coldest phase at around 43.5 ka. It can thus be argued that the IUP population had a low density. Slimak[9] pointed out that the Neronian lithic tradition dated to 54 ka was directly linked to AMHs. This pushes back the arrival of modern humans in Europe by 10 ka and implies the incursion of modern humans into Neandertal territory. He pointed out that the relationships between the Neronian and the Levantine IUP question the validity of the concepts that define the first AMH migrations and the nature of the first Upper Palaeolithic in western Eurasia. It is suggested that a trans-Mediterranean technical connection existed which indicate three distinct waves of AMHs expansion into Europe between 55–42 ka. The study of Slimak[9] is an analysis of spatial connections over approximately 4000 km from east to west using stone tool typology and an integration of a very small number of spatially isolated sites into three successive dispersal waves. While the details of this study need to be further scrutinized, this attempt is inspirational and has the greatest gain of raising the awareness that several colonization attempts of AMHs in Europe before the Aurignacian can be expected, which apparently failed.
From the modelling perspective, we are limited by the lack of IUP data for the derivation of the HEP. Unless we have a sufficient understanding of the interconnections between the IUP and AUR populations, it will be difficult to parameterize them without controversies. Nevertheless, we carried out three IUP-related experiments (Experiments BS8, BS9 and BS10 listed in Supplementary Tab. 2 as a first attempt to model the impact of IUP populations on the Aurignacian dispersal. These model results must be interpreted in conjunction with the assumptions made.

The IUP sites considered in the IUP experiments are listed in the Supplementary Tab. 3.

In Experiment BS8, we assumed human presence at 50 ka at four IUP sites, namely, Bacho Kiro, Temnata Dupka, Bohunice and Zlatý kůň. The population density is assumed to be initially Gaussian distributed in space, specified as

$$\rho(r) = \rho_0 exp(-r^2/2\sigma_r^2)$$

with r being the distance from the IUP site, ρ_0 the peak value of population density (set to 0.025 P km^{-2}) and σ_r the standard deviation (set to 50 km). Since the number of IUP sites known today is too small for deriving a HEP model, we used that for AUR-P1 to estimate the HEP for the IUP. We also lowered the cultural carrying capacity from 5 P km^{-2} for the AUR to 3 P km^{-2} for the IUP, reduced the diffusion coefficient by a factor of two and the birth rate from 1% for the AUR to 0.1% for the IUP. Embedded in this somewhat arbitrary choice is the assumption that the IUP techno-complexes may be less sophisticated as the AUR. Note that the model has an internal logic for human

dispersal and population growth. If the population growth is low, then rapid human dispersal is unlikely. The population growth rate depends not only on climate and environment conditions, but also on the cultural carrying capacity, an external parameter representing the human capacity to harness resources. While IUP and AUR were modern human techno-complexes, they probably had different carrying capacities and hence different population growth rates. The simulation ran for the period 50 to 42 ka (when the humans of the AUR firmly settled central Europe). Shown in Supplementary Fig. 7a, b, c, and d are the simulated IUP population densities at 48, 45, 43 and 42 ka, respectively. The results suggest that the humans of the IUP were mainly confined to the Balkan region, with a dispersal preference in the south/southeast direction, opposite to that of the AUR. The highest population density reached at 42 ka was 0.018 P km^{-2} due to the low cultural carrying capacity and low birth rate we assumed. Shown in Supplementary Fig. 7e and f are the suppositions of the IUP and AUR populations. This experiment suggests that the IUP human groups could not compete with a large inflow of humans of the AUR and went either extinct or assimilated to the latter.

For BS9, we assumed human presence at 50 ka at the same four IUP sites as for BS8 but used all parameters identical as for AUR-P1. As in Supplementary Fig. 7, shown in Supplementary Fig. 8a, b, c and d are the simulated IUP population densities for 48, 45, 43 and 42 ka, respectively, and in Supplementary Fig. 8e and f the suppositions of the IUP and AUR populations. In this scenario, humans of the IUP would have expanded first into the Balkan region and Italy and later into Anatolia, with a dispersal preference in the direction opposite to that of the AUR. They would have then occupied west Europe between 48 and 45 ka. By 45 ka, they would have achieved a stable settlement in a territory like that of the AUR-P1 at 41 ka (see Fig. 1), including the Mediterranean coast of Spain. While the results of BS9 leaves open the debate whether the AUR culture on the pan-European scale represented an east-to-west propagation or an east-and-west co-evolution rooted in the IUP, they cannot be verified with observational data and need to be further scrutinized.

In BS10, we assumed human presence at 50 ka at three Uluzian sites in Italy (Fumane, Grotta di; Cavallo, Grotta del; and Castel Civita) and two Châtelperronian sites in France (Grotte du Renne; Trou de la Chèvre), as listed in Supplementary Tab. 3. All other parameters are as for BS8. Again, shown in Supplementary Fig. 9a, b, c and d are the simulated IUP population densities at 48, 45, 43 and 42 ka, respectively. The results suggest that the humans originated from the Châtelperronian sites were mainly propagating to the western part of France, while the humans originated from the Uluzian sites were mainly propagating in Italy and with a dispersal preference eastward along the Mediterranean coast, opposite to that of the AUR. The population density reached at 42 ka was low due to the low cultural carrying capacity and low birth rate we assumed. Shown in Supplementary Fig. 9e and f are the superpositions of the IUP and AUR populations. Also, this experiment suggests that the IUP human groups, due to the much lower population density,

could not compete with a large inflow of humans of the AUR and went either extinct or assimilated to the latter.

3.2 Validation Strategy

The OWM consists of two basic components: (1) the HEP model that combines climate/environment data and archaeological site data for HEP estimation, using logistic regression, and (2) the human dispersion model that simulates the population dynamics and predicts the human population density. The output of the HEP model is an essential input for the human dispersal model. Accordingly, the strategy for the validation of the OWM also consists of two components: (1) whether the HEP model correctly reproduces the human existence potential, and (2) whether the OWM correctly reconstruct the human dispersal history. As already mentioned in the main text, the validation of the HEP model is not the main issue here, as this has been done in our previous publications[1,8,10] and some of the results are shown in Section 3.3.

The OWM results can be validated in the following ways.

- Population density estimates: high-quality and independent estimates of the population density of humans of the AUR would provide a valuable validation of the OWM reconstructed population density. Unfortunately, such estimates are very few (as cited in Section 2 Results and Discussion) and have very large uncertainties;
- Arrival time: The OWM has its internal logic which determines the speed of human dispersal, as this is collectively determined by several factors, including the temporal and spatial patterns of the HEP, the drift and diffusion processes, the reproduction rate and the population pressure. Thus, the arrival time of humans offers a powerful validation of the model performance. A handful of the archaeological sites are dated and have been used to validate the OWM-simulated arrival time, as shown in Fig. 2;
- Consistency with archaeological understanding: An important validation is to use expert knowledge to examine the consistency of the model results with the archaeological understanding achieved independently. For example, based on the distribution and chronology of the archaeological sites in the Balkan and Upper Danube areas, Mellars[4] hypothesized that the AUR expansion started somewhere in Turkey and progressed in a major event during the AUR-P1. The latter author proposed approximate expansion routes (his Fig. 2). Shao et al.[1] estimated the Best Potential Paths based on the HEP patterns, which are consistent with the suggestions of Mellars[4]. As a validation strategy, we examined whether the model-predicted expansion routes, now quantified using population fluxes, are consistent with the studies[1,4], as shown in Fig. 4;
- Self consistency: a key feature of the OWM is to learn about the HEP from archaeological data, which in turn serves as a driver for population dynamics. From this perspective, an area of high HEP is expected with high probability to be settled sometime in the migration history, while

the OWM simulation shows whether, when and how the potential may be realized. Obviously, a poorly functioning model may produce results other from the expectations. Thus, without circular arguments, a measure of the OWM goodness is whether the archaeological sites are correctly reflected in the model-predicted population density. Fig. 10a shows the probability distribution function of population density at all AUR-P1 sites for 41 ka when the AUR-P1 settlement reached a maximum and in Fig. 10b that at all AUR-P2 sites for 38 ka when the AUR-P2 settlement reached a maximum. As seen, the population density at most of the archaeological sites are high, confirming the self-consistency of the OWM.

4 Supplementary References

All plots are made with the python packages Matplotlib[11] and Cartopy[12]. State borders are made with Natural Earth (Free vector and raster map data at naturalearthdata.com).

The climate reconstruction data, the Human Existence Potential, and the Our Way Model results[13] used or generated in this study have been deposited in the figshare database https://doi.org/10.6084/m9.figshare.26174980. The data behind all figures can be found in this entry as well.

The code of the human dispersal model and for all figures of the results[14] have been deposited in the figshare database https://doi.org/10.6084/m9.figshare.26203691.

[1] Shao, Y. *et al.* Human-existence probability of the aurignacian techno-complex under extreme climate conditions. *Quaternary Science Reviews* **263**, 106995 (2021). https://doi.org/10.1016/j.quascirev.2021.106995 .

[2] Wegener, C. *Development and Application of a Climate-driven Human Dispersal Model.* Ph.D. thesis, Universität zu Köln (2021). URL https://kups.ub.uni-koeln.de/54602/.

[3] Timmermann, A. & Friedrich, T. Late Pleistocene climate drivers of early human migration. *Nature* **538** (7623), 92–95 (2016). https://doi.org/10.1038/nature19365 .

[4] Mellars, P. A. Palaeoanthropology: The earliest modern humans in europe. *Nature* **479**, 483–485 (2011). https://doi.org/10.1038/479483a .

[5] Hajdinjak, M. *et al.* Initial Upper Palaeolithic humans in Europe had recent Neanderthal ancestry. *Nature* **592** (7853), 253–257 (2021). https://doi.org/10.1038/s41586-021-03335-3 .

[6] Bergström, A., Stringer, C., Hajdinjak, M., Scerri, E. M. & Skoglund, P. Origins of modern human ancestry. *Nature* **590** (7845), 229–237 (2021). https://doi.org/10.1038/s41586-021-03244-5 .

[7] Higham, T. *et al.* The timing and spatiotemporal patterning of Neanderthal disappearance. *Nature* **512** (7514), 306–309 (2014). https://doi.org/10.1038/nature13621 .

[8] Klein, K. *et al.* Assessing climatic impact on transition from neanderthal to anatomically modern human population on iberian peninsula: a macroscopic perspective. *Science Bulletin* **68** (11), 1176–1186 (2023). https://doi.org/10.1016/j.scib.2023.04.025 .

[9] Slimak, L. The three waves: Rethinking the structure of the first upper paleolithic in western eurasia. *PLOS ONE* **18** (5), 1–20 (2023). URL https://doi.org/10.1371/journal.pone.0277444. https://doi.org/10.1371/journal.pone.0277444 .

[10] Klein, K. *et al.* Human existence potential in europe during the last glacial maximum. *Quaternary International* **581-582**, 7–27 (2021). https://doi.org/10.1016/j.quaint.2020.07.046 .

[11] Hunter, J. D. Matplotlib: A 2d graphics environment. *Computing in Science & Engineering* **9** (3), 90–95 (2007). https://doi.org/10.1109/MCSE.2007.55 .

[12] Met Office. *Cartopy: a cartographic python library with a Matplotlib interface.* Exeter, Devon (2010 - 2015). URL https://scitools.org.uk/cartopy.

[13] Wegener, C. *Supplementary data collection HEP and OWM* (2024). URL https://doi.org/10.6084/m9.figshare.26174980.

[14] Wegener, C. *Supplementary code collection HEP and OWM* (2024). URL https://doi.org/10.6084/m9.figshare.26203691.
